# The combination of an oxygen-dependent degradation domain-regulated adenovirus expressing the chemokine RANTES/CCL5 and NK-92 cells exerts enhanced antitumor activity in hepatocellular carcinoma

**DOI:** 10.3892/or.2012.2217

**Published:** 2012-12-28

**Authors:** JIANG LI, HUI LIU, LINFANG LI, HONGPING WU, CHUNHONG WANG, ZI YAN, YING WANG, CHANGQING SU, HUAJUN JIN, FUPING ZHOU, MENGCHAO WU, QIJUN QIAN

**Affiliations:** Laboratory of Viral and Gene Therapy, Eastern Hepatobiliary Surgical Hospital and Institute, The Second Military Medical University, Shanghai 200438, P.R. China

**Keywords:** RANTES, adenovirus, hepatoma, gene therapy, adoptive immunotherapy

## Abstract

Oncolytic adenoviruses are modified based on adenovirus serotype 5 (Ad5), which belongs to subgroup C and depends on Coxsackie-adenovirus receptor (CAR) to recognize target cells. However, expression of CAR is generally low or lost in certain tumors including hepatocellular carcinoma (HCC). By contrast, CD46 is highly expressed in various types of malignant tumor cells. Therefore, we constructed an adenovirus vector expressing the human RANTES/CCL5 gene regulated by oxygen-dependent degradation domain (ODD) and analyzed its antitumor effects *in vitro* and *in vivo*. The human RANTES/CCL5 gene was fused with ODD by PCR and the recombinant oncolytic adenovirus containing RANTES-ODD, SG511-CCL5-ODD, was constructed by the Gateway system, which infected cells by binding CD46. Viral replication experiments were performed to evaluate the selective replication ability of SG511-CCL5-ODD. RANTES expression was determined by ELISA. The chemotactic test was used to analyze the ability of the expressed RANTES to recruit NK92 cells. The antitumor effects of SG511-CCL5-ODD were examined in HCC xenografts in nude mice. A chimeric oncolytic adenovirus, SG511-CCL5-ODD, was constructed successfully. Cells infected with the recombinant virus were able to express RANTES selectively in different environments controlled by ODD and the expressed RANTES was able to recruit NK92 cells by its chemotactic effect *in vitro* and improve the anticancer immune response in HCC xenografts in nude mice. The chimeric adenovirus SG511-CCL5-ODD highly expressed the RANTES-ODD fusion gene in the hypoxia of HCC under the control of the ODD and effectively attracted NK92 cells and a high number of immunocytes. These factors had complementary advantages and, in combination, exerted enhanced antitumor efficacy.

## Introduction

T cells provoke the antitumor immune response; therefore, T cell-mediated immunotherapy is regarded as a crucial approach in cancer treatment. The adoptive cell transfer (ACT) combined with lymphodepletion has led to clinical tumor suppression in 50–70% of patients with metastatic melanoma (1). However, the large number of activated and expanded T cells required in the laboratory (more than 1×10^10^) makes ACT a costly and labor-intensive treatment (2–4).

Owing to the marked effects in the treatment of metastatic melanoma and the evident impact on cancer regression in approximately 50% of patients, development of ACT therapy utilizing autologous tumor-infiltrating lymphocytes (TIL) is gaining momentum (5,6). In addition, although some ACT-treated patients achieved long-term survival, the majority experienced cancer relapse (7). Therefore, strategies aimed at improving the migration of T cells into tumor sites are likely to enhance the efficacy of ACT therapy.

Immune cells trafficking to inflammation sites are usually affected by chemokines. Through chemotaxis, cells that express appropriate chemokine receptors can migrate along with a chemokine gradient to specific tissues or infection sites (8). Chemokines also play an important role in T cell-mediated antitumor immune responses (9–11). A variety of immunocytes have strong chemotactic effects after being regulated upon the activation of normal T-cell expressed and secreted RANTES/CCL5 that chemotactically attracts a huge number of immunocytes to tumor tissues to exert antitumor efficacy (12–17).

As a result of immune response unable to eradicate tumor cells, cancer develops. There may be mechanisms significantly involved in tumor development, which allow tumor cells to escape immune surveillance from natural killer (NK) and other immune cells (18–20). NK cells represent distinct subsets of lymphoid cells with innate immune functions (21). NK cells are derived from the bone marrow, they circulate in the blood and then become activated by cytokines or upon encountering target cells expressing ligands for NK cell receptors (22). NK cells provide one of the first lines of defense against virus-infected and tumor cells. In general, no immune receptor gene rearrangement occurs in them and their cytotoxicity is not MHC-restricted. NK cells are able to mediate the spontaneous killing of various tumor cells without prior sensitization. To date, not all NK cell lines can be established from patients with large granular lymphoma (23) and, with regard to the *in vitro* tumoricidal properties, these NK cell lines show considerable differences, although some of them have been shown to maintain cytotoxicity (23,24).

Isolated from peripheral lymphocytes of a non-Hodgkin’s lymphoma patient in 1992 (25), NK-92 was successfully set up as a natural anti-IL-2 dependent cell line, with similar functional characteristics as human NK cells. Research demonstrated that NK-92 cells showed high cytotoxic activity against tumor cell lines from leukemia (26) and malignant melanoma (27). Additionally, *in vivo* application of NK-92 was tested in immune deficiency (SCID) mouse models, xenografted with patient-derived leukemia (T-ALL, AML) (24) or human malignant melanoma cell lines (27). Tumor burden was reduced or undetectable and the survival of mice was significantly improved. NK-92 cells are constantly being developed for adoptive immunotherapy in cancer treatment and have entered clinical trials (28).

The most widely-used oncolytic adenoviruses in cancer therapy are based on adenovirus serotype 5 (Ad5), which belongs to subgroup C and Coxsackie-adenovirus receptor (CAR) on target cells are necessary for successful transduction (29,30). However, expression of CAR is generally low or lost in the process of the malignant progression of certain tumors, including hepatocellular carcinoma (HCC) (31–33), limiting the transduction of Ad5-based vectors in some tumor cells and resulting in impaired antitumor effects (31). By contrast, CD46, a cellular receptor for Ad11 (subgroup B) (34), is highly expressed in various types of malignant tumor cells including HCC cells (35–38). Therefore, to address the issue of CAR-dependent cell entry, novel fiber chimeric adenoviral vectors have recently been designed by switching the knob and shaft of the Ad5 fiber to those of the Ad11 fiber that can recognize the abundant CD46 receptor on tumor cells (39). Previous studies have indicated that viral entry and antitumor activity could be greatly improved by 5/11 fiber chimeric oncolytic adenoviruses compared with Ad5-based viruses (40,41). Nevertheless, the tumor-suppressing capacity of 5/11 fiber chimeric oncolytic adenoviruses in HCC have yet to be fully explored.

Genetic modification of signal pathways promoting cell growth and survival leads to the emergence of tumor cells, whose expansion depends on nutrient supply. Oxygen limitation is crucial for controlling angiogenesis, glucose metabolism, survival and tumor spread. This effect is orchestrated by hypoxia-inducible factor (HIF), a main transcriptional factor in nutrient stress signal. Variations in oxygen tension (*p_O2_*) are not directly sensed by HIF but by a class of 2-oxoglutarate-dependent and iron-dependent dioxygenases which belong to the family of non-haem oxidizing enzymes. Due to strict control of *p_O2_* to the activity, these enzymes are the true oxygen-sensing molecules controlling the hypoxic response. Two types of oxygen sensors control HIF action; the first are known as the prolyl hydroxylase domain (PHD) proteins. PHDs hydroxylate two prolyl residues (P402 and/or P564) in the human HIF-1α region considered the oxygen-dependent degradation domain (ODD). This HIF-α modification specifies rapid interaction with the tumor-suppressor protein von Hippel-Lindau (VHL), a component of an E3 ubiquitin ligase complex. As a result, HIF-α subunits are marked with polyubiquitin chains driving them to destruction by the proteasomal system. ODD is closely related to the rapid degradation of intracellular proteins (42). Some proteins with ODD display instability under normoxia, but stability under hypoxia. Hypoxia exists within most solid tumors (43–46). Hence, as a regulatory element, the translated ODD can lead to a concentration of certain proteins at a specific high level.

Therefore, we constructed a chimeric adenovirus SG511-CCL5-ODD carrying RANTES-ODD fusion gene. We hypothesized that in hypoxia of HCC, RANTES is highly expressed under the control of ODD and attracts a large number of immunocytes to tumor tissues to exert antitumor efficacy. NK92 cells were also administered by tail vein injection and they can be attracted to the tumor site by RANTES. In the HCC tumor site, SG511-CCL5-ODD and NK92 cells jointly exerted an enhanced antitumor activity.

## Materials and methods

### Cell lines and culture

Human primary HCC cell lines Hep3B and SK-Hep-1 and human normal fibroblast cell lines BJ were purchased from the American Type Culture Collection (ATCC, Manassas, VA, USA). Human HCC cell lines, SMMC-7721, BEL-7404 and BEL-7405 and human normal hepatocellular cell lines L02 were obtained from the Institutes of Cell Biology, Chinese Academy of Sciences (Shanghai, China). The human embryonic kidney (HEK) 293 cell line was obtained from Microbix Biosystem, Inc. (Toronto, ON, Canada). NK-92 was purchased from the American Type Culture Collection, and was maintained in α medium (Gibco BRL, Gaithersburg, MD, USA) supplemented with 2 mM L-glutamine, 0.2 mM i-inositol, 20 mM folic acid, 0.1 mM 2-mercaptoethanol, 12.5% fetal bovine serum (FBS) and 12.5% horse serum containing 100 U human IL-2 (Sigma). L02, SMMC-7721, BEL-7404 and BEL-7405 were maintained in RPMI-1640 with 10% FBS and HEK293, SK-Hep-1 and Hep3B were maintained in Dulbecco’s modified Eagle’s medium (Gibco BRL) in a 5% CO_2_ atmosphere at 37°C, containing 10% FBS. BJ was maintained in modified Eagle’s medium (Gibco BRL) in a 5% CO_2_ atmosphere at 37°C, containing 10% FBS. All media were supplemented with 100 U/ml penicillin and 100 μg/ml streptomycin. Hypoxic conditions (1% O_2_) were achieved with a three-gas incubator (Thermo Scientific).

### Construction of adenovirus

pCMV-SPORT6 vector containing human RANTES gene was purchased from Proteintech Group, Inc. Ad5/F11 mosaic adenovirus plasmids, pPE3-F11-ccdB, pSG500 (47), pPE3-F11, were modified in our lab. pPE3-F11 was constructed by switching the knob and shaft of the pBHGE3 (Microbix Biosystem) fiber to those of the Ad11 fiber. pPE3-F11-ccdB was inserted ccdB (ccdB is negative selection in *E. coli* following recombination and transformation) into E3 area of pPE3-F11. We used the primers 1–4 as follows: primer 1: CGGAATTCACCATGAAGGTCTCCG CGGCAG; primer 2: GCTAACATCTCCAAGTCTAAGCT CATCTCCAAAGAG; primer 3: GTCATCATCCATTGGGAT ATAGGGAGCTAACATCTCCAAG; primer 4: CGGGAT CCCTATAACTGGAAGTCATCATCCATTG. The RANTES gene and ODD domain were fused and amplified by overlapping PCR, in which the restriction endonuclease sites, *Eco*R I and *Bam*H I, were introduced into the upstream and downstream of the fused gene. The PCR fragment was inserted into pENTR12 (Invitrogen) and pDC315 (Microbix). After confirming by sequencing, the generated plasmids were labeled as pENTR12-CCL5-ODD, pDC315-CCL5-ODD. pENTR12-CCL5-ODD was recombined with adenovirus backbone plasmid pPE3-F11-ccdB by Gateway recombination in DH5α; the generated adenovirus backbone vector was identified by restriction endonuclease digestion and labeled pPE3-F11-CCL5-ODD. Adenovirus shuttle plasmid pSG500 with E1a controlled by the hTERT promoter and E1b controlled by HRE was transfected together with pPE3-F11-CCL5-ODD into HEK293 cells by Lipofectamine 2000 reagent, to generate a tumor-selective proliferating adenovirus, SG511-CCL5-ODD. Plasmid pDC315-CCL5-ODD was transfected together with pPE3-F11 into HEK293 cells by Lipofectamine 2000 reagent, to generate a tumor-selective proliferating adenovirus, AD5/11-CCL5-ODD. The viral titers of SG511-CCL5-ODD and AD5/11-CCL5-ODD were measured with the tissue culture 50% infective dose (TCID_50_) method.

### In vitro viral replication assay

Cells in contact-inhibition phase and cancer cells in log-phase were seeded in 6-well plates and infected with the recombinant adenoviruses at a multiplicity of infection (MOI) of 5.0 plaque-forming units (pfu)/cell. Cells were then washed twice with PBS and incubated at 37°C, 1% O_2_ for 0, 48 or 96 h. Cell lysates were prepared with three cycles of freezing and thawing. Serial dilutions of the lysates were titered in HEK293 cells, with the TCID_50_ method, normalized with that at the beginning of infection and reported as multiples.

### ELISA for CCL5 expression

In the *in vitro* experiments, SMMC-7721, BEL-7404 and BEL-7405 were seeded in 6-well plates at a density of 5×10^5^ cells per well. To investigate the CCL5 expression in hepatoma cells, SMMC-7721, BEL-7404 and BEL-7405 were infected with SG511-CCL5-ODD at an MOI of 5 pfu/cell in normoxia and hypoxia, respectively. At 72 h after infection, the supernatants were collected to detect CCL5 expression by ELISA (R&D).

### In vitro cell viability assay

3-(4,5-dimethylthiazol-2-yl)-2, 5-diphenyltetrazolium bromide (MTT) assay was performed to determine cell viability at various viral MOIs. Cells were plated at a density of 1×10^4^ in 96-well plates (Gibco); 24 h later, cells were infected with SG511-CCL5-ODD at a wide range of MOI from 0.001 to 100 pfu/cell. After 7 days of incubation, cell viability was measured by MTT assay using the non-radioactive cell proliferation kit (Roche Molecular Biochemicals), according to the kit protocol, and the spectrophotometrical absorbance of the samples was read on a microtiter plate reader at 570 nm in a reference wavelength of 650 nm. The percentage of cell survival was calculated using the formula: Cell survival (%) = (A value of infected cells/A value of uninfected control cell) × 100%. Eight replicating samples were obtained at each MOI and each experiment was performed at least three times.

### Transwell chamber chemotactic assay

Serum-free MEM/αM (containing 2×10^5^ cell/200 μl) was added to the upper compartment of the invasion chamber. Conditioned medium (1 ml) was added to the lower compartment of the invasion chamber. The lower compartment of the invasion chamber of the control and the standard group were supplemented with 1 ml serum-free MEM/αM and 1 ml containing 1 ng/ml RANTES serum-free MEM/αM, respectively. The invasion chambers were incubated at 37°C for 24 h, and the inserts and cells on the upper side of the filter were then removed. Cells that invaded the underside of the filter were counted. Each experiment was repeated three times. The values obtained were calculated by averaging the total number of cells from triplicate determinations.

### Animal experiments

BALB/c nude mice (nu/nu) were purchased from the Shanghai Experimental Animal Center, Chinese Academy of Sciences. SMMC-7721 cancer cells in log phase were subcutaneously injected into the right flanks of mice (1×10^7^ per mouse). Two weeks later, the tumor xenografts were established, 40 mice were randomly assigned to four groups (SG511, AD5/11-CCL5-ODD, SG511-CCL5-ODD and the control group, n=10 mice/group). Tumors were then injected with 100 μl control buffer or 2×10^8^ pfu of viruses. The injections were repeated every other day for five times, with a total dosage of 1×10^9^ pfu.

Other SMMC-7721 tumor xenograft models were established as mentioned above to evaluate the combined antitumor efficacy of SG511-CCL5-ODD and NK-92. The mice were divided randomly into four groups: I (SG511-CCL5-ODD), II (NK-92), III (SG511-CCL5-ODD92) and control (10 mice/group). Group I was given a total of 1×10^9^ pfu viruses five times by intratumoral injection, once every other day. The mice in group II received intravenous inoculations of 2.5×10^7^ NK-92 cells five times, once every other day. Group III received intratumoral injection of 2×10^8^ pfu viruses in 100 μl viral preservation solution and at the indicated time after injection, 5×10^6^ NK-92 in 200 μl of PBS were administered by intravenous inoculation, the animals received a series of five doses of viruses and NK-92 cells. In the control group, 100 μl viral preservation solution [10 mM Tris-HCl (pH 8.0), 2 mM MgCl_2_, 4% sucrose] per mouse per time was injected intratumorally. Tumor volume was estimated with the formula: (maximal diameter) × (perpendicular diameter)^2^ × 0.5. All animal experiments were performed according to the Guidelines for the Institutional Animal Care and Use Committee of The Second Military University (Shanghai, China).

### Immunohistochemistry

Tumor samples were formalin-fixed and paraffin-embedded and successive sections for H&E staining and immunohistochemistry were prepared. CCL5-expressing cells were assessed by staining with a goat anti-human CCL5 (R&D) followed by incubation with a biotin-conjugated rabbit anti-goat IgG and a horseradish peroxidase (HRP)-conjugated streptavidin (Southern Biotech). NK-92 cells were assessed by staining with a mouse anti-human CD56 (Santa Cruz) followed by incubation with a biotin-conjugated goat anti-mouse IgG and a horseradish peroxidase-conjugated streptavidin. The positive reaction was visualized with 3′,3′-diaminobenzidine. The sections were examined by two independent investigators for qualitative and semiquantitative analysis.

### Statistical analysis

Experiments were performed three times and data are shown as the means ± SD. Student’s t-test was used to interpret the significance of differences between every two groups. ANOVA for repeated measurement experiments was conducted to compare the tumor growth over time between the treated groups and the control group in the animal experiment. P<0.05 was considered to indicate statistically significant differences.

## Results

### Adenovirus selectively proliferates and mediates RANTES expression in HCC cells

**Figure 1:**
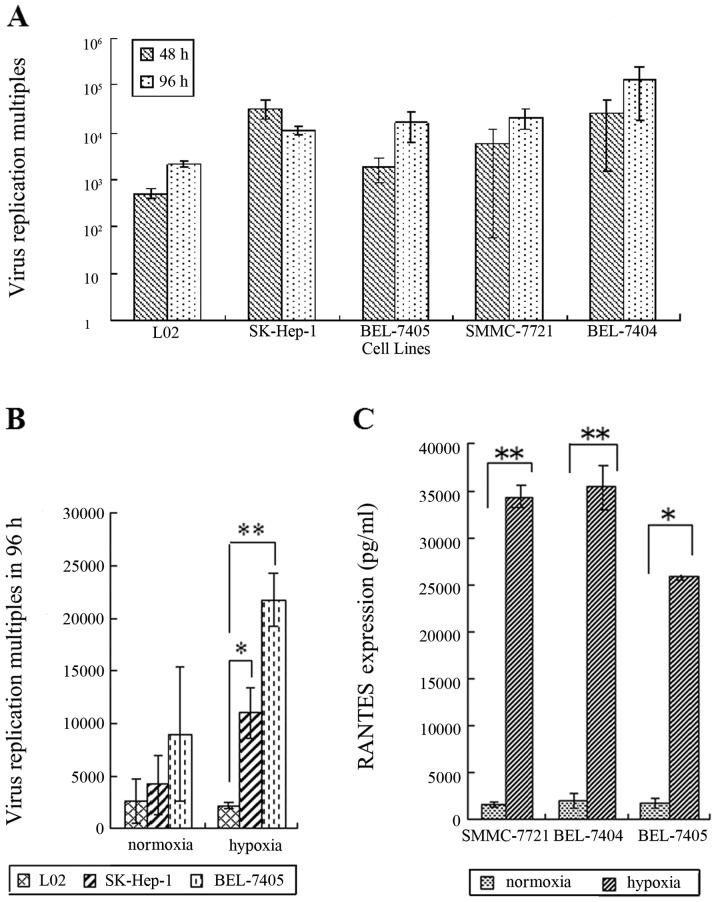
SG511-CCL5-ODD selectively replicates and mediates high expression of RANTES in HCC cells. (A) Selective replication ability of SG511-CCL5-ODD ^*^p<0.05; ^**^p<0.01. (B) Selective replication ability of SG511-CCL5-ODD in different proportions of oxygen in 96 h. ^*^p<0.05; ^**^p<0.01. (C) Protein expression of the *RANTES* gene in different proportions of oxygen. ^*^p<0.05; ^**^p<0.01.

The Ad5/F11 adenovirus showed a geometrical multiple proliferation in liver cancer cell lines, particularly in BEL-7404 with the proliferation multiple of 250,000 times in hypoxia at 96 h, demonstrating the virus has strong proliferation ability in cancer cells (Fig. 1A). The proliferation multiple of SG511-CCL5-ODD in the normal cell line was very low, showing that SG511-CCL5-ODD has weak proliferation ability in normal cells (Fig. 1B).

With the viral proliferation, Ad5/F11 chimeric adenoviruses express RANTES protein at high levels in SMMC-7721, BEL-7404 and BEL-7405 cells, reaching up to 35427.3 pg/ml in BEL-7404 cells in hypoxic conditions. Protein expression of the *RANTES* gene in hypoxia was much higher than that in normoxia in hepatoma cell lines; ODD can effectively regulate RANTES protein expression (Fig. 1C, P<0.05).

### Cytotoxic specificity of SG511-CCL5-ODD by MTT assay

**Figure 2:**
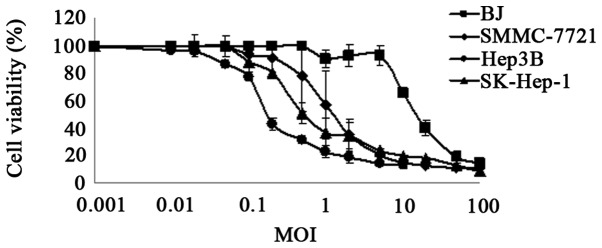
Selective oncolytic effect of SG511-CCL5-ODD on HCC cell lines. At MOI = 10, the cell viability was <30% in HCC cells, but >60% in normal cells when infected with SG511-CCL5-ODD.

MTT assay was performed to characterize the specificity of SG511-CCL5-ODD in tumor cells. As shown in Fig. 2, SG511-CCL5-ODD caused significant cytolysis in the Hep3B, Sk-Hep-1 and SMMC-7721 cell lines at a MOI of 0.1 pfu/cell, 0.5 pfu/cell and 1 pfu/cell, respectively. However, normal cells infected with SG511-CCL5-ODD showed a >50% cell viability at an MOI of 10 pfu/cell, suggesting that >10–100-fold of SG511-CCL5-ODD were needed to kill half of the normal fibroblast cells compared with liver cancer cells (Fig. 2).

### Transwell chamber chemotactic assay

**Figure 3:**
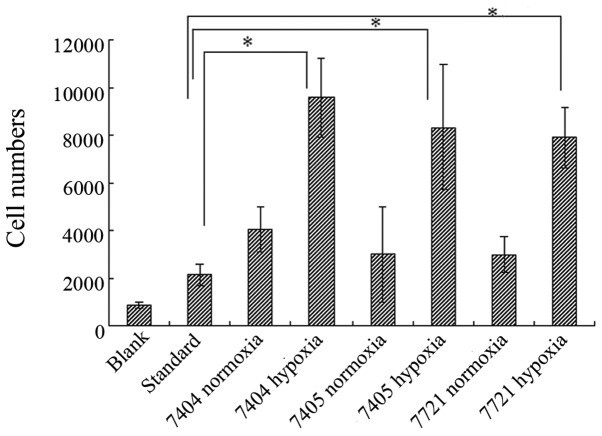
Chemotaxis experiment of CCL5. Chemotactic test results indicated the expressed RANTES can recruit NK-92 cells. In hypoxia, chemotactic capability was more effective than in normoxia (P<0.05).

The chemokine experimental results showed that, compared with the control group and the standard group which contained RANTES protein at 1 ng/ml in the lower compartment of the invasion chamber, the RANTES expressed by Ad5/F11 chimeric adenovirus presented the chemotactic activity for NK-92 cells. In hypoxia, the chemotactic effect was higher than in normal oxygen conditions (P<0.05) (Fig. 3).

### Ad5/F11 chimeric adenovirus-mediated RANTES expression exerts antitumor potency in HCC xenograft models

**Figure 4:**
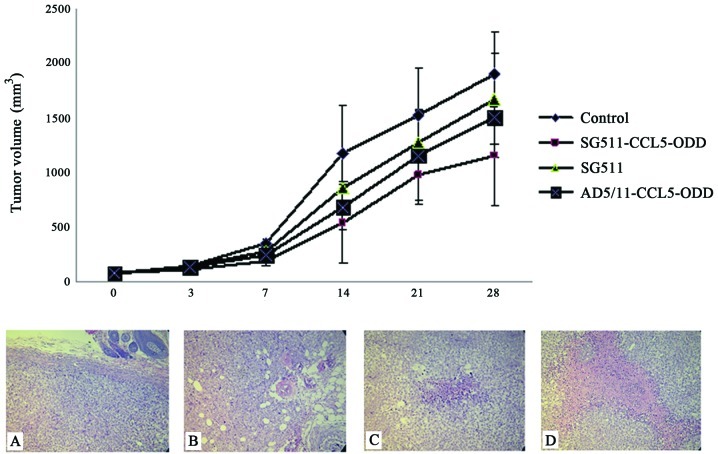
Antitumor efficacy of RANTES in SMMC-7721 tumor xenografts. Mice were given five intratumoral injections to introduce viruses into SMMC-7721 xenografts, one injection every other day with 2×10^8^ plaque-forming units/dose/mouse. The potent antitumor effect was shown in all virus-treated groups. SG511-CCL5-ODD showed the best antitumor growth effect. The antitumor effect of SG511-CCL5-ODD and AD5/11-CCL5-ODD was stronger than that of SG511. (A-D) Pathological examination of tumor specimens, magnification ×200. H&E staining showed wide areas of necrosis in tumor tissues of the SG511-CCL5-ODD-treated group, but cancer cells grew abundantly in the control group; (A) control; (B) SG511; (C) AD5/11-CCL5-ODD; (D) SG511-CCL5-ODD.

In the SMMC-7721 xenograft models, SG511-CCL5-ODD intratumor treatment significantly inhibited tumor growth compared with the control group from day 14 post-treatment (P<0.001). Both SG511-CCL5-ODD and AD5/11-CCL5-ODD treatment groups showed stronger antitumor activity than the SG511 group from day 14 post-treatment (P<0.05) (upper panel, Fig. 4). Mice were sacrificed after the observation period. Tumors were collected and examined pathologically by H&E staining. Several large necrotic regions were found in tumors from each group, especially in the SG511-CCL5-ODD-treated group. In the control group, however, cancer cells grew unhindered with only small focal areas of necrosis (Fig. 4A-D).

### Antitumor efficacy of SG511-CCL5-ODD combined with NK-92

**Figure 5:**
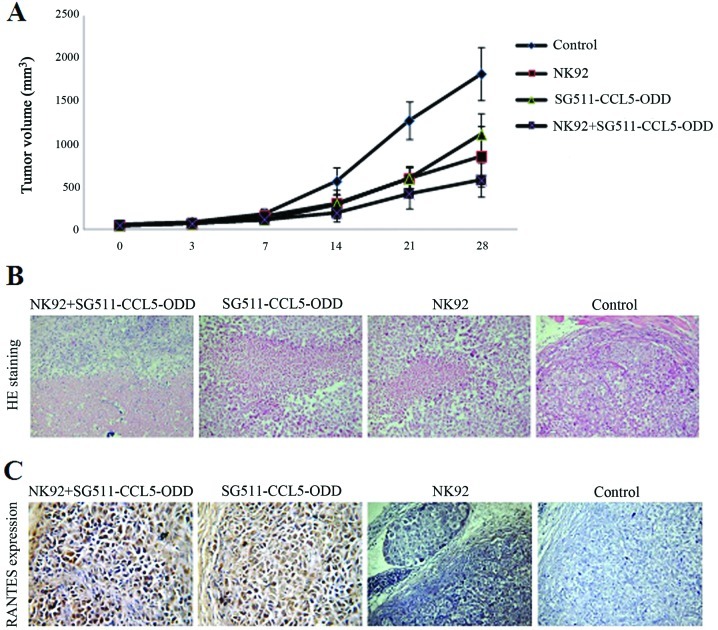
Antitumor efficacy of RANTES combined with NK92 in SMMC-7721 tumor xenografts. (A) Mice were given five intratumoral injections to introduce SG511-CCL5-ODD viruses into SMMC-7721 xenografts, one injection every other day with 2×10^8^ plaque-forming units/dose/mouse or given five intravenous inoculations of 5×10^6^ NK-92 cells five times, once every other day. The potent antitumor effect was shown in all virus-treated and/or NK92-treated groups. The combined group of SG511-CCL5-ODD and NK92 showed the best antitumor growth effect. The antitumor effect of NK92 was stronger than that of SG511-CCL5-ODD. (B) H&E staining pathological examination of tumor specimens, magnification ×200. H&E staining showed wide areas of necrosis in tumor tissues of the SG511-CCL5-ODD combined with the NK92-treated group, but cancer cells grew abundantly in the control group. (C) The hepatocytes were positive for RANTES protein in mice of the NK92+SG511-CCL5-ODD and the SG511-CCL5-ODD group injected with SG511-CCL5-ODD; magnification ×200.

The antitumor efficacy of SG511-CCL5-ODD combined with NK-92 was evaluated on SMMC-7721 tumor xenografts established in nude mice. Significant reduction of tumor volume was observed in all the treatment groups, with tumor inhibition rates of 51.19, 53.28 and 34%, respectively, for Group I (SG511-CCL5-ODD), Group II (NK-92), Group III (SG511-CCL5-ODD92), when compared with the control group (P<0.05) on day 14 after treatment, and 46.15, 46.42 and 32.7% on day 21, 61.04, 46.81 and 31.46% on day 28, respectively (Fig. 5A).

Mice were sacrificed 28 days later by cervical dislocation. All tumor samples were examined histologically using H&E staining (Fig. 5B) and immunohistochemical staining for RANTES (Fig. 5C). In the control group, cancer cells grew abundantly with small foci of necrosis. In the groups treated with replicating viruses or (and) NK-92 cells, several wide areas of necrosis were observed. There were many necrotic foci in tumor tissues of the SG511-CCL5-ODD combined NK-92 groups. Around the necrotic areas, most cancer cells were positive for RANTES expression, but there were no cancer cells positive for RANTES in the NK-92 and control groups.

## Discussion

RANTES (CCL5) is an 8-kDa cytokine, with clear chemotactic activity to the cells which are involved in the immune/inflammatory response, such as lymphocytes, monocytes/macrophages. RANTES can also regulate the function of effector cells. Chemotaxis of RANTES relies on the concentration of RANTES. Therefore, the aggregation of RANTES can enhance its role in chemotaxis. By increasing the local concentration of RANTES, the district can attract the cells by the concentration gradient of the cytokine.

This study successfully constructed a selective replication adenovirus SG511-CCL5-ODD, in which the hTERT promoter drives the E1a gene and the hypoxia response promoter controls the E1b gene. This adenovirus is specifically replicated in tumor cells. ELISA assay was used to detect RANTES protein expression in tumor cells infected with SG511-CCL5-ODD. We found RANTES protein expression was different in both normoxic and hypoxic conditions, when in the same MOI and at the same time. Expression in hypoxia was significantly higher than in normoxia (P<0.05), confirming that adenovirus SG511-CCL5-ODD in hypoxic conditions has a stronger ability of gene expression. The results proved the RANTES gene under the control of ODD, RANTES protein in normal oxygen conditions is partially degraded.

The chemotaxis test is a method used to test the effectiveness of chemokine. Compared with the control, we found the virus in liver cancer cells could highly express RANTES protein and had a strong chemotactic effect to NK-92 cells. The supernatant of liver cancer cells which was infected by SG511-CCL5-ODD was more effective to chemotaxis NK-92 cells in hypoxic conditions. The RANTES protein was confirmed as a strong chemotaxis agent for NK-92 cells. High concentrations of local tumor chemokine RANTES protein attract immune cells around the tumor tissue to maintain a high concentration of immune cells, which is key to cancer biological therapy.

In the liver cancer xenograft model, significant tumor growth inhibition was demonstrated in all the treatment groups compared with the control group. From day 14 after treatment, the tumor volume in the mice treated with SG511-CCL5-ODD combined with NK-92 was suppressed more than that in the mice with any other treatment. After a 4-week treatment, when compared with the control group, the tumor volume was reduced by 68.54 and 53.19%, respectively, in Group III (SG511-CCL5-ODD92) and Group II (NK-92), and Group III (SG511-CCL5-ODD92) showed more tumor repression than Group II (NK-92) (P<0.01). These results indicate that all treatments [including I (SG511-CCL5-ODD), II (NK-92) and III (SG511-CCL5-ODD92)] inhibited tumor growth and III (SG511-CCL5-ODD92) is the most efficient agent among them. Therefore, immune cell-viral biotherapy is superior to simple immunotherapy and virotherapy.
